# Difficulties in implementation of the project 'HUPE against sepsis': speaking of people who watch

**DOI:** 10.1186/cc12919

**Published:** 2013-11-05

**Authors:** Sérgio da Cunha, Mário Castro Álvares Perez, Elisabete Novello Ferreira, Luana Ferreira de Almeida, Eliane Passos Pereira Assumpção, Paulo Vieira Damasco, Jorge da Silva Motta, Rogério Marques de Souza, Viviane Silva e Silva, Elizabeth de Andrade Marques, Vagner Ismerim Lobão, Irene de Souza e Silva, Ana Alice de A Triani, Jessica Bernardes Almeida Borges da Silva, Julio Cesar Delgado Correal, Catherine Valdez, Jessica Oliveira

**Affiliations:** 1 Department of Clinical Medicine, College of Medical Ciences, Rio de Janeiro State University, Rio de Janeiro, Brazil; 2 Nursing Department, Pedro Ernesto University Hospital, Rio de Janeiro State University, Rio de Janeiro, Brazil; 3 Department of Internal Medicine, College of Medical Ciences, Rio de Janeiro State University, Rio de Janeiro, Brazil; 4 Medical Coordination, Pedro Ernesto University Hospital, Rio de Janeiro State University, Rio de Janeiro, Brazil; 5 Nursing Coordination, Pedro Ernesto University Hospital, Rio de Janeiro State University, Rio de Janeiro, Brazil; 6 Bacteriology, Pedro Ernesto University Hospital, Rio de Janeiro State University, Rio de Janeiro, Brazil; 7 Central Laboratory, Pedro Ernesto University Hospital, Rio de Janeiro State University, Rio de Janeiro, Brazil; 8 Pharmacy, Pedro Ernesto University Hospital, Rio de Janeiro State University, Rio de Janeiro, Brazil; 9 School of Medical Sciences, Rio de Janeiro State University, Rio de Janeiro, Brazil; 10 School of Medical Sciences, Rio de Janeiro State Federal University, Rio de Janeiro, Brazil

## Introduction

The project 'HUPE against sepsis' seeks to emphasize the importance of early recognition of sepsis, in order to accelerate the implementation of measures associated with decreased mortality for severe sepsis. It is therefore important that the professionals involved in healthcare are attentive to quick detection of signs and symptoms associated with the condition. The objective was to identify the difficulties for the implementation of the protocol advocated by the Surviving Sepsis Campaign and adopted by the project 'HUPE against sepsis'.

## Materials and methods

The study was conducted in clinical medical and surgical wards, DIP, general duty, cardiac and ICUs of the Pedro Ernesto University Hospital (HUPE), totaling 11 inpatient units. In January 2013, a questionnaire was applied to doctors, nurses and nurse technicians, including effective, contractors and residents. This instrument contained closed questions, professional profile and was related to the topic in question.

## Results

Fifty-one professionals participated in the study: 22 (43%) medical staff and 29 (57%) nurses. Of these, 12 were medical residents, and eight were nursing residents, all in the first year (approximately 78% of workers investigated). Most physicians (55%), 38% of nurses and 40% of nurses claimed to have greatest difficulty administering the first dose of antibiotics within up to 1 hour after the diagnosis. About 45% of doctors and 31% of nurses also reported difficulty in the distribution of materials to acquire the sepsis kit (which contains materials for deep venous puncture, invasive hemodynamic monitoring and collecting blood cultures). Physicians (41%) and nurses (40%) still reported as a problem going to the pharmacy to get the first dose of the antibiotic. Other limiting factors were also appointed: obtaining the vesical catheterization of delay (for hourly diuresis control); rapid identification of severe sepsis; printed data record of the protocol; samples of blood culture for aerobic; and peripheral venous access puncture.

## Conclusion

The difficulties pointed out by the professionals investigated are common and include factors that prevent the correct and early implementation of the protocol, be they of institutional and/or professional responsibility. Seeking solutions to the problems raised allows a targeting of future actions to be developed, among them the constant updating and training of professionals involved in assistance for the inpatients investigated. This allows, also, the search for better institutional infrastructure appropriate to meeting the demands of the patient with severe sepsis.

